# Comparative Quantitative Studies on the Microvasculature of the Heart of a Highly Selected Meat-Type and a Wild-Type Turkey Line

**DOI:** 10.1371/journal.pone.0170858

**Published:** 2017-01-24

**Authors:** Salah Al Masri, Maria Kattanek, Kenneth C. Richardson, Hafez Mohamed Hafez, Johanna Plendl, Hana Hünigen

**Affiliations:** 1 Institute of Veterinary Anatomy, Department of Veterinary Medicine, Freie Universität Berlin, Berlin, Germany; 2 College of Veterinary Medicine, School of Veterinary and Life Sciences, Murdoch University, Murdoch, Australia; 3 Institute of Poultry Diseases, Department of Veterinary Medicine, Freie Universität Berlin, Berlin, Germany; Worcester Polytechnic Institute, UNITED STATES

## Abstract

In this study the macroscopic and microscopic structure of the heart of a fast growing, meat-type turkey line (British United turkeys BUT Big 6) and a wild-type turkey line (Canadian Wild turkey) were compared. At 8 and 16 weeks of age, 10 birds of each genotype and sex were sampled. The body mass and heart mass of the meat-type turkey both increased at a faster rate than those of the wild-type turkey. However in both turkey lines, the relative heart mass decreased slightly with age, the decrease was statistically significant only in the male turkeys. Furthermore meat-type turkeys had a significantly (p < 0.01) lower relative heart mass and relative thickness of the left ventricle compared to the wild-type turkeys of the same age. The wild-type turkeys showed no significant change in the size of cardiomyocytes (cross sectional area and diameter) from 8 weeks to 16 weeks. In contrast, the size of cardiomyocytes increased significantly (p < 0.001) with age in the meat-type turkeys. The number of capillaries in the left ventricular wall increased significantly (p < 0.001) in wild-type turkeys from 2351 per mm^2^ at the age of 8 weeks to 2843 per mm^2^ at 16 weeks. However, in the meat-type turkeys there were no significant changes, capillary numbers being 2989 per mm^2^ at age 8 weeks and 2915 per mm^2^ at age 16 weeks. Correspondingly the area occupied by capillaries in the myocardium increased in wild-type turkeys from 8.59% at the age of 8 weeks to 9.15% at 16 weeks, whereas in meat-type turkeys this area decreased from 10.4% at 8 weeks to 9.95% at 16 weeks. Our results indicate a mismatch in development between body mass and heart mass and a compromised cardiac capillary density and architecture in the meat-type turkeys in comparison to the wild-type turkeys.

## Introduction

The worldwide growing demand for poultry meat has resulted in pressure on breeders, nutritionists and growers to increase the growth rate of birds, feed efficiency, and size of breast muscle. Today, turkeys are marketed in about half the time and at about twice the body weight compared to 50 years ago [1]. These changes are due mainly to the high heritability of body weight and body meat composition [2]. This kind of selection has lowered the capacity of modern growing birds to respond to stressors, like the responses to heat stress in their environment [3] and some believe it has resulted in the failure of several organ and body systems because of the increased metabolic demands required for extremely rapid increases in body mass [4, 5].

Undesirable traits including many circulatory disorders, such as ascites, aortic rupture, spontaneous cardiomyopathy (round heart), and cardiomyopathy causing sudden death, each accompanied by a lowered muscle production and/or high mortality, in turkeys have arisen, presumably due to the stress induced by having such rapid growth [6–9]. As an example an enigma over recent times is the occurrence, often with no obvious causal agent, of perirenal hypertrophic cardiomyopathy in flocks of rapidly growing, heavy, male turkeys. Here mortality, usually 2–10%, is most common between 8–18 weeks, the period of most metabolic stress associated with rapid muscle development [5]. Julian [10] suggests that in turkeys with noninfectious cardiovascular disorders the cardiomyocytes can respond to significant changes in blood pressure and volume as well as to a lack of oxygen in only a very limited way, that is to enlarge to meet the increased demand placed on them.

According to Schmidt et al. [11] in a comparison of modern broiler turkey lines with heritage turkey lines, the apparent lower physiological capacity to accommodate increasing skeletal muscle volume of modern broilers can be explained partly by their low relative heart mass. They reported that the hearts of the heritage line birds [UIUC] grew at a rate of 7 mg/g of bird, whereas the hearts of the modern broiler birds [Ross] grew at a rate of 5 mg/g of bird. When heritage and modern broiler birds of equivalent weight were compared, the UIUC hearts were larger than those of the Ross lineage.

The postnatal growth of the heart in domestic birds has been studied predominantly from the standpoint of normal development [11] and pathology [7, 12]. Likewise the vascular system morphology of turkeys has been studied primarily from a gross pathology point of view [5, 13].

There is little information on the influence of age, gender and genetics on the development of the heart and the cardiac capillary architecture of turkeys. A comparative study was carried out to examine differences in heart structure over the critical growth periods of a highly selected meat-type and a wild-type turkey line, to elucidate the probable relations between genetic selection for rapid growth and cardiovascular diseases in turkeys. The aim was to compare morphological and microscopic architectural features of the heart and the myocardial capillaries of Canadian wild turkeys to that of a highly genetically selected meat-type domestic turkey line during their rapid growth period between 8 and 16 weeks of age.

## Animals, Materials and Methods

This study involving turkey handling and treatments was carried out in accordance with German animal welfare law. The protocol was approved by State Office of Health and Social Affairs Berlin (LaGeSo Reg. Nr. 0218/07).

### Animals and husbandry

Forty wild-type turkeys (Wild Canadian Turkeys) (20 male and 20 female) obtained from a wildlife park (Wild- und Freizeitpark Ostrittrum, Germany), as well as forty meat-type turkeys from a highly selected line (British United Turkeys BUT Big 6) (20 male and 20 female) obtained from a commercial grow-out farm (Gut Jäglitz GMBH & Co. Agrar KG, Roddahn, Germany) were selected as day-old-chicks. This study was approved by the responsible Animal Care Committee (Landesamt für Gesundheit und Soziales Berlin, Germany).

Wild-type and meat-type birds were housed separately in two groups under the same husbandry conditions (10 birds/6.5 m^2^), in the Institute of Poultry Diseases, Department of Veterinary Medicine, Freie Universität Berlin. All birds were fed a commercial pellet diet (Ströh Hobbersdorf, Pansdorf, Germany) using a three stage feeding system. This consisted of starter feed (type 015) for weeks 1 to 6, then growers feed (type 016) from week 7 to 12, and finishers feed I (type 017) from week 13 onwards. All birds were allowed ad libitum access to food and water. The study ended on week 16.

### Sample collection and processing

**Fig 1:**
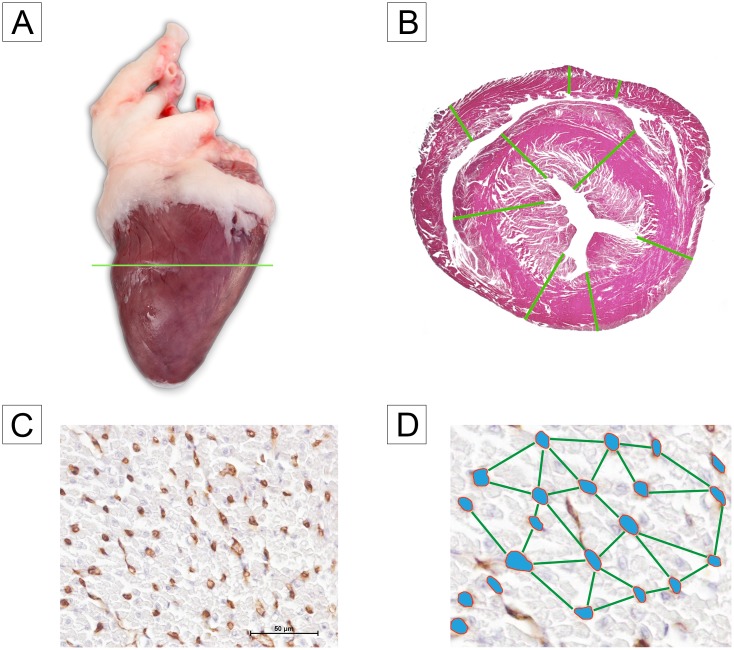
(A) Position of the heart sample cross section, note fat deposits in coronary sulcus, (B) the locations of the measurements taken for each ventricular structure, (C) Arachis hypogaea Lectin stained blood capillaries (brown), and (D) stylized plot showing how intercapillary distance was determined.

At 8 and 16 weeks of age, 10 birds of each genotype and sex were sampled. Live body masses were measured to an accuracy of 0.1 kg using a mechanical scale (Sartorius, Göttingen, Germany). Then the birds were killed according to Germany’s animal welfare standards by stunning and exsanguination. Immediately after a bird’s death the heart was dissected free from the carcass and weighed to an accuracy of 0.001 g on an electronic laboratory balance (Sauter-Cumulus, Freiburg, Germany). Then a one-centimeter-wide cross section sample was dissected from the midpoint between base and apex of the heart and prepared for morphometric examination (Fig 1A). Here the specimens were washed in 0.9% sodium chloride solution and fixed in phosphate buffered formalin (4%, pH 7, 24 h, room temperature). They were then dehydrated in a graded series of ethyl alcohol and embedded in paraffin wax. Serial sections were cut at 5 μm and stained either with hematoxylin and eosin (H&E) or with the capillary endothelial marker Arachis hypogaea lectin (Peanut agglutinin, PNA) according to Aescht et al. [14].

### Morphometric analysis

From each sample the thickness of both ventricular walls and the interventricular septum was measured in section stained with H&E using an image analyzing system, Nis Elements (Nikon). Thicknesses of each ventricular structure were measured at three different locations at a magnification of 100x (Fig 1B). Structure thicknesses are defined as the average value of the 3 measurements.

In addition, from each bird, 25 cardiomyocytes from the left ventricular wall were measured at a magnification of 100x and the average value was calculated. Here the size of the cardiomyocytes was determined by measuring the cross sectional area and the diameter of the cardiomyocytes at the level of the nucleus.

To determine the density of the blood capillaries, for each bird, the area of interest was defined by randomly choosing 5 visual fields under 400 × magnification (Fig 1C and 1D). Only midmyocardial regions with transversely oriented myocytes and circular capillaries were used. The area of each field of view was 34124.89 μm^2^. The following parameters in lectin stained slides were then measured using an image analyzing system NIS-Elements (Nikon):

- number of blood capillaries per mm^2^
- number of the cardiomyocytes per mm^2^
- intercapillary distance: 25 intercapillary distances for each sample were measured, starting with the capillary located nearest to the center of the field. The shortest distance from the outer circumference of this capillary wall was drawn manually to the neighboring capillaries and from these radially outwards until 25 distances were measured—and the average value was determined
- percentage of the area occupied by capillaries

### Statistical analysis

Statistical analyses were performed using SPSS for Windows v. 20 software (SPSS, Chicago, IL). The mean values of the collected parameters were compared between the groups using the Mann—Whitney U-test, p < 0.05 was considered statistically significant. Results are presented as box-and-whisker plots (median, interquartile range, and range).

## Results

### Macroscopic examination of the turkey hearts

#### Topography and general morphology of the heart

Topography and morphology of the heart were similar in both turkey lines and in both age groups. The heart is located in the cranial third of the visceral cavity. It is conically shaped and flattened dorsomedially. It is enclosed within the fibrous pericardial sac that is connected apically to the hepatopericardial and sternopericardial ligaments and thereby attached to the dorsal surface of the sternum. The heart lies in the median line, with its long axis almost parallel to that of the body. The base is attached indirectly to the spine by dorsally traversing intersegmental arteries arising segmentally from the descending aorta. The coronary sulcus, separating the atria from the ventricles typically had fat deposits (Fig 1A). The apex rests in the anterior part of the median fissure of the liver and is oriented towards the right side.

Anatomic pathology examination after slaughter revealed no signs of disease.

#### Body weight, heart weight and relative heart weight

Male turkeys of both lines and at both slaughter ages had a significantly higher body mass than females. In the meat-type turkeys average body mass increased from 3.62 kg at week 8 to 13.79 kg at week 16 in males and in females from 3.07 kg to 10.11 kg. In wild-type turkeys at the same times, male body mass increased from 1.09 kg to 3.35 kg and in females from 0.86 kg to 2.47 kg (Table 1). The body mass of male meat-type birds increased by a factor of 3.8 whilst in females the factor was 3.3. In comparison, the body mass of wild-type turkeys increased by a factor of 3.1 for males and 2.9 for females.

**Table 1:** Mean ± SD of body weight, heart weight, relative heart weight and the thickness and the relative thickness of the left ventricular wall, the interventricular septum and the right ventricular wall of meat-type versus wild-type turkeys.

Parameters	Age (weeks)	Wild-type turkey				Meat-type turkey			
		Female (n = 10)		Male (n = 10)		Female (n = 10)		Male (n = 10)	
		Mean	SD	Mean	SD	Mean	SD	Mean	SD
Body mass (kg)	8	0.86	0.06	1.09	0.18	3.07	0.19	3.62	0.45
	16	2.47	0.21	3.35	0.21	10.11	0.44	13.79	0.97
Heart mass (g)	8	5.20	0.50	7.14	1.34	14.60	1.51	17.85	1.92
	16	14.78	2.25	19.74	2.22	45.51	4.42	61.09	7.38
Relative heart mass (%)	8	0.61	0.04	0.65	0.06	0.48	0.06	0.50	0.04
	16	0.60	0.06	0.59	0.09	0.45	0.04	0.44	0.05
Left ventricular wall [mm]	8	3.17	0.51	3.14	0.34	4.56	0.28	4.87	0.54
	16	4.17	0.69	4.38	1.17	6.30	0.70	6.94	0.84
Interventricular septum [mm]	8	3.68	0.76	4.01	0.64	4.84	0.47	5.00	0.74
	16	4.15	0.58	4.79	1.26	6.01	0.72	6.17	0.59
Right ventricular wall [mm]	8	1.40	0.20	1.47	0.21	1.25	0.31	1.49	0.61
	16	1.60	0.36	1.66	0.60	1.62	0.47	1.78	0.29
Relative thickness of left ventricular wall [mm/kg]	8	3.72	0.51	2.90	0.57	1.49	0.16	1.37	0.26
	16	1.54	0.27	2.08	1.00	0.62	0.07	0.50	0.06
Relative thickness of septum wall [mm/kg]	8	4.34	0.85	3.65	0.55	1.59	0.22	1.40	0.29
	16	1.54	0.25	2.38	1.40	0.60	0.07	0.45	0.06
Relative thickness of right ventricular wall [mm/kg]	8	1.66	0.26	1.35	0.24	0.41	0.11	0.41	0.13
	16	0.59	0.14	0.82	0.46	0.16	0.05	0.13	0.02

The average heart mass of female and male, 8-week-old meat-type turkeys, was 14.6 g and 17.85 g, respectively (Table 1). In the wild-type turkeys of the same age these were 5.2 g and 7.14 g respectively. In the 16-week-old female and male meat-type turkeys the average heart mass was 45.51 g and 61.09 g (3.4 and 3.1 fold increases) and that of the wild-type turkeys at the same age was 14.78 g and 19.74 g (2.8 and 2.9 fold increases) respectively.

**Fig 2:**
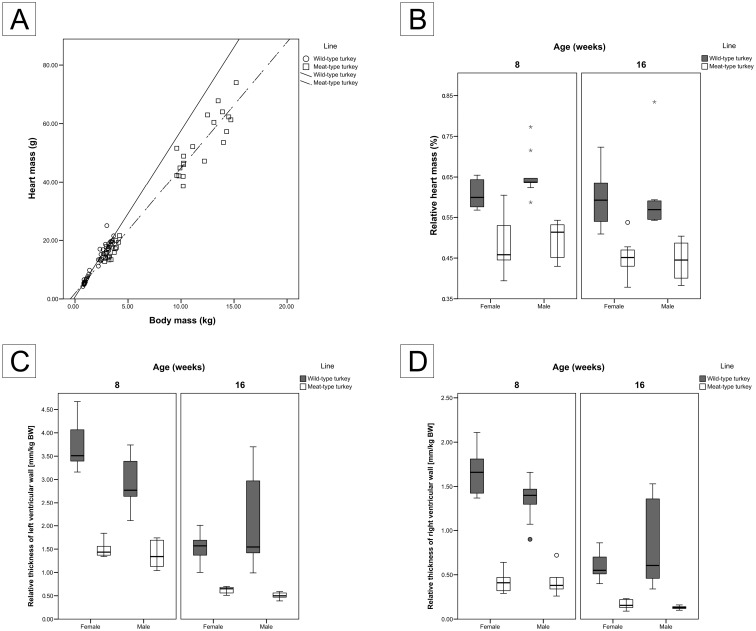
Growth of heart in the wild- and meat-type turkeys. (A) Plot of heart mass (g) versus total body mass (kg), (B) relative heart mass (%) versus age in weeks, (C) relative thickness of the left ventricular wall (mm/kg) and (D) relative thickness of the right ventricular wall (mm/kg). Graphs B, C, and D are presented as boxplots with medians and whiskers with maximum 1.5 of the interquartile range (IQR), (o) suspected outliers and (☆) outliers.

In both turkey lines, the relative heart mass decreased slightly with age, the decrease was more prominent and statistically significant in the male turkeys than in the females (Table 1 and Fig 2B). In both turkey lines, the relative heart mass of the male turkeys decreased by 0.06% with age (p ≤ 0.006 and 0.016 for wild-type and meat-type turkeys, respectively).

Furthermore, meat-type turkeys had a significantly (p < 0.01) lower relative heart mass compared to the wild-type turkeys of the same age and gender groups (Table 1 and Fig 2A and 2B).

#### Thickness and relative thickness of the ventricular walls

The thicknesses of the left ventricle wall increased with age in both turkey lines. The meat-type turkeys with values of 4.56–6.94 mm were significantly thicker than that of the wild-type turkeys with values of 3.14–4.38 mm (Table 1). The thicknesses of the right ventricle wall increased with age in both turkey´s lines. There were no statistically significant differences in the thicknesses of the right ventricular walls of the two turkey lines (Table 1).

The relative thickness of the left ventricle wall decreased with age in both turkey´s lines. The decrease in the relative thickness of the left ventricle wall was statistically significant in all groups except for the male wild-type turkeys (p ≤ 0.06), (Table 1 and Fig 2C). The relative thickness of the left ventricle wall of male wild-type turkeys decreased by a factor of 0.18 whilst in females the factor was of 0.59. In comparison, the relative thickness of the left ventricle wall of meat-type turkeys decreased by a factor of 0.64 for males and 0.65 for females.

Moreover, meat-type turkeys had significantly (p < 0.001) lower relative thickness of the left ventricle wall compared to the wild-type turkeys of the same age and gender groups.

Within the same line and age groups, and except the 16-week-old wild-type turkeys, the relative thickness of the left ventricle was greater in females than in males (Table 1 and Fig 2C). But the differences were statistically significant only in the 8-week-old wild-type turkeys and in the 16-week-old meat-type turkeys (p ≤ 0.021 and 0.005, respectively).

The development pattern of the right ventricle wall was similar to that of the left ventricle wall. The relative thickness of the right ventricle wall decreased with age by factors of 0.64 (female) and 0.39 (male) in the wild-type turkeys and by factors of 0.61 and 0.68 in meat-type turkeys, respectively (Table 1 and Fig 2D). The only significantly gender related difference was found in the 8-week-old wild-type turkeys (p = 0.034). Here the average relative thicknesses of the right ventricle wall were 1.66 and 1.35 for the female and the male 8-week-old wild-type turkeys, respectively.

The development pattern of the septum was similar to that of the left ventricle wall.

The ratio of the ventricular walls (left:septum:right) was 2.4:2.7:1 for the wild-type turkeys, and 3.7:3.6:1 for the meat-type turkeys.

### Microscopic examination of the turkey hearts

The general microscopic morphology of the heart was similar in both turkey lines and showed no differences due to age or gender. The slender, elongated cardiomyocytes were arranged into small groups by loose connective tissue containing a rich network of capillaries (Fig 1C and 1D).

#### Size of the cardiomyocytes

**Fig 3:**
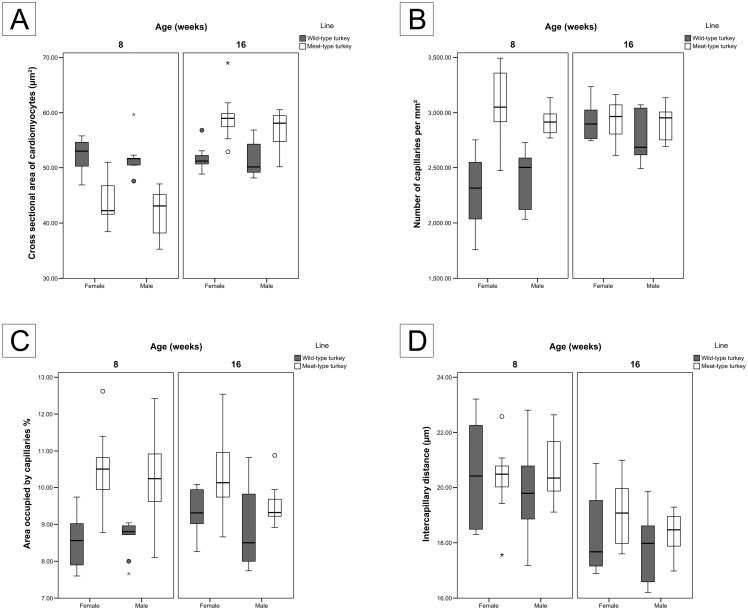
Size of the cardiomyocytes and capillary measures in the wild- and meat-type turkeys. Each graph is a gender-based comparison. (A) cross sectional area of the cardiomyocytes (μm^2^), (B) number of blood capillaries per mm^2^, (C) percentage area occupied by capillaries and (D) intercapillary distances (μm). All graphs are presented as boxplots with medians and whiskers with maximum 1.5 of the interquartile range (IQR), (o) suspected outliers and (☆) outliers.

The average cross sectional area and the diameter of the cardiomyocytes for all the birds examined were 51.2 μm^2^, and 8.05 μm, respectively (Table 2). The wild-type turkeys showed no significant change in the size of cardiomyocytes from 8 weeks to 16 weeks (Table 2 and Fig 3A). However, the size of cardiomyocytes (cross sectional area and the diameter) increased significantly (p < 0.001) with age in the meat-type turkeys (Table 2 and Fig 3A). Both size parameters of the cardiomyocytes were significantly smaller in the meat-type line than in the wild-type line at 8 weeks (p < 0.001), however they were larger at 16 weeks (p < 0.001) (Table 2).

**Table 2:** Mean ± SD of the cross sectional area and the diameter of cardiomyocytes, number of capillaries, area occupied by capillaries, intercapillary distance and the number of the cardiomyocytes per capillary of meat-type versus wild-type turkeys.

Parameters	Age (weeks)	Wild-type turkey				Meat-type turkey			
		Female (n = 10)		Male (n = 10)		Female (n = 10)		Male (n = 10)	
		Mean	SD	Mean	SD	Mean	SD	Mean	SD
Cross sectional area of cardiomyocytes (μm^2^)	8	52.34	3.04	51.86	3.25	44.13	4.02	41.96	4.19
	16	51.75	2.13	51.47	3.05	59.20	4.25	56.92	3.45
Diameter of cardiomyocytes (μm)	8	8.16	0.24	8.12	0.25	7.49	0.34	7.30	0.37
	16	8.11	0.16	8.09	0.24	8.68	0.31	8.51	0.26
Number of capillaries per mm^2^	8	2289.92	334.03	2411.84	263.80	3064.36	328.02	2914.33	118.6
	16	2920.19	171.68	2766.07	212.63	2922.54	187.12	2906.71	164.6
Area occupied by capillaries %	8	8.54	0.74	8.64	0.48	10.56	1.01	10.24	1.22
	16	9.29	0.63	9.00	1.18	10.36	1.14	9.53	0.56
Intercapillary distance (μm)	8	20.48	1.96	19.83	1.60	20.32	1.27	20.73	1.17
	16	18.29	1.49	17.89	1.27	19.11	1.14	18.41	0.69
Number of the cardiomyocytes per capillary	8	7.80	1.19	7.42	0.99	6.72	0.80	7.42	0.84
	16	6.03	0.37	6.43	0.47	5.21	0.33	5.51	0.57

There was no significant gender related differences within the same line and age groups.

#### Capillary density

The average number of capillaries in the left ventricular wall of the heart increased significantly (p < 0.001 for female and 0.013 for male) in wild-type turkeys from 2351 at the age of 8 weeks to 2843 per mm^2^ at 16 weeks, (Table 2 and Fig 3B). In contrast, in the meat-type turkeys there were no significant changes, capillary numbers being 2989 per mm^2^ at age 8 weeks and 2915 per mm^2^ at age 16 weeks. Within the same age and gender groups, the number of capillaries was greater in the meat-type turkeys than in the wild-type turkeys, but the difference was statistically significant only in the 8-week-old birds (p < 0.001).

The area occupied by capillaries in the myocardium (gender averaged) increased in wild-type turkeys from 8.59% at the age of 8 weeks to 9.15% at 16 weeks, whereas in meat-type turkeys they were 10.4% at 8 weeks and 9.95% at 16 weeks (Table 2 and Fig 3C). Furthermore, the area occupied by capillaries in the 16-week-old males of the meat-type turkeys, decreased significantly from 10.24% to 9.53 (p ≤ 0.049) (Table 2 and Fig 3C).

The difference between the two turkey lines was statistically significant in both age groups (p < 0.001 for 8 weeks and 0.035 for 16 weeks). However, at 16 weeks the difference in the male turkeys was statistically insignificant (p ≤ 0.226) (Table 2 and Fig 3C).

The only difference in the intercapillary distance between the groups was age related. The intercapillary distance decreased in both groups from ~20 μm at 8 weeks to ~18 μm at 16 weeks (Table 2 and Fig 3D).

In both turkey lines, the number of the cardiomyocytes per capillary decreased significantly with age. In the 8-week-old turkeys, there was no significant difference in the number of the cardiomyocytes per capillary between the both turkey lines. At this age, one capillary supplied on average 7.61 and 7.07 cardiomyocytes in wild-type turkeys and meat-type turkeys, respectively. At the age of 16 weeks, the difference between the turkey lines was significant (p < 0.001 for female and 0.002 for male), where in the 16-week-old turkeys in meat-type birds there were 5.36 and in wild-type birds 6.23 cardiomyocytes supplying each capillary, representing a decrease of 24% and 18% respectively.

## Discussion

During domestication the growth rate as well as the feed conversion rate of modern turkey lines has improved considerably. However the genetic selection for high meat production has altered the balance between the relative growth of the locomotor/musculoskeletal system and that of the organs of domestic turkeys [15, 16] as well as of domestic chickens [11]. The selection for rapid muscle growth in domestic chickens is associated with cardiovascular problems [7] that are believed to be due to a genetically linked predisposition [9, 17, 18]. This is supported by indications of a propensity for spontaneous cardiomyopathy being found in the genome of turkeys [19]. To elucidate the probable linkages between genetic selection for rapid muscle growth and cardiovascular diseases in turkeys, a comparative study was undertaken to reveal differences in heart structure over the critical growth periods of 8 and 16 weeks of a highly selected meat-type and a wild-type turkey line.

It has been well established that the relative heart mass is an ideal parameter in comparative morphology to assess the cardiovascular performance of a species. Hartman [20] reported on 291 avian species covering 64 families, that the relative heart mass of birds varies from 0.2% to 2.4%. Here species having values as low as 0.2% such as tinamous are species that have low energy demands because in their daily activity budget walking is their main locomotor activity. This contrasts to species such as swifts that have a high relative heart mass of 1.61%, presumably due to their main locomotor activity being rapid, prolonged flying in their daily activity budget. The turkeys of our study had a relative heart mass of about 0.54%. This corresponds well with the earlier studies where bird species relying primarily on bipedal locomotion rather than flying have low relative heart masses.

In the present study, meat-type turkeys in both age groups had a significantly lower relative heart mass compared to wild-type turkeys of the same age. This is most likely a reflection of the more active lifestyle of wild-type turkeys to the sedentary lifestyle of modern highly selected meat-type turkeys.

Our study supports the findings of Pannwitz et al. [21] who reported a relative heart mass of 0.42% for 17 week old female and 24 week old male meat-type BUT turkeys. Similarly in the study of Nestor et al. [15] 16 weeks old meat-type turkeys had a relative heart mass of 0.39% for males and 0.34% for females. In our study, both lines of turkeys showed a higher relative heart mass than reported for meat type turkeys by Nestor et al. [15] and by Pannwitz et al. [21].

Whilst the influence of genetic selection is believed to be the main influence on heart mass, husbandry conditions may have an effect on relative heart mass. For example Millan et al. [22] found that farm-bred red-legged partridges had a 12% lighter, relative heart mass than did wild partridges at the same age. In this study the farm partridges had not undergone any genetic selection, however, improved feeding regimens were probably responsible for the different visceral organ development of the farmed birds. The birds of our study were kept in groups of 10 birds on an area of 6.5 m^2^ and had considerably more space to move than turkeys in modern conventional farms. This fact may be one of the reasons for the higher relative heart mass of our meat-type animals compared to the data of Pannwitz et al. [21] and Nestor et al. [15] on meat-type turkeys.

There are conflicting reports in the literature on the influence of gender on the heart mass of birds. A lower relative heart mass in male African Muscovy ducks (1.1%) versus 1.5% in females was reported by Teguia et al. [23], However Boulianne et al. [24] reported a higher relative heart mass in male turkeys (0.33%) compared to females (0.28%). The turkeys of the present study exhibited only minor differences between male and female birds.

Age related differences in the relative heart mass were found in a modern meat-type chicken line (Ross 708), with a significant decrease in the relative heart mass commencing at 2 weeks old that continued to decrease from then until slaughter [11]. This did not occur in a matching heritage line (UIUC) [11]. In the turkeys of the present study only a minor decrease in the relative heart mass was found between 8 and 16 weeks of age.

Our morphometric data show similar thicknesses of the left ventricular wall and the interventricular septum and these were much thicker in the meat-type than in the wild-type turkeys. Moreover, these parameters increased greatly with age, 40.4% in meat-type turkeys and 35.4% in wild-type turkeys. Thus, meat-type turkeys have an accentuated growth of the left ventricle. This supports the findings of Anatskaya et al. [25] who found non—proportionally faster growth in the left ventricle compared to other heart chambers during the last stage of proliferative heart growth of turkeys. The increase in the left ventricular wall thickness was not due to an increase in the number of the cardiomyocytes. It was due to an increase of the size of the cardiomyocytes during postnatal growth of the avian heart as described in 31 different avian species by Anatskaya et al. [25]. Given that the increase in cell size is coupled with larger diffusion distances for oxygen and nutrients of these cells, this may be a possible limiting factor in heart performance.

In addition, the number and size of the capillaries supplying the cardiomyocytes as well as the diffusion distances between capillaries and the cardiomyocyte’s cytoplasm are important descriptive parameters for the oxygen and nutrient supply of the myocardium. Quantitative studies of the formation of the capillary network in the myocardium of birds are rare. Michel et al. [26] reported a relationship of 1 capillary to 4 cardiomyocytes in domestic chickens, 1:6 in domestic ducks and 1:9 in pheasants. In the mammalian heart the number of capillaries per mm^2^ was reported as about 2000–4000 in the human [27, 28] and rat [29, 30], 5000 capillaries in sheep [31] and 2500 in the Yak [32]. These results indicate the large range of these allied parameters among different species.

The cardiac capillary density of the turkeys in the present study supports the numbers reported above. Between 8 and 16 weeks of age, the area occupied by capillaries increased slightly in the wild-type birds, but decreased, also slightly, in the meat-type turkeys. In contrast, the size of the cardiomyocytes remained constant in wild-type but increased significantly between 8 and 16 weeks of age in the meat-type birds. The intercapillary distance decreased with age in both turkey types, from ~20 μm at 8 weeks to ~18 μm at 16 weeks. Hence the diffusion pathway for oxygen and nutrients to the cardiomyocyte cytoplasm is reduced with increasing age.

In future studies using either vascular casting or high-resolution imaging technique like micro-CT may clarify the three-dimensional architecture of the microvasculature of the turkey heart.

Avian cardiomyocytes are characterized by their slender shape and their arrangement into groups of 5–8 cells [33]. Domestic chickens, ducks and pheasants all have a cell diameter ranging from 3.5–6.3μm [26, 34, 35]. In our study, both lines of turkeys had cardiomyocyte diameters of 7–8 μm, i.e. somewhat larger than those reported above.

The higher growth rate of meat-type turkeys is associated with a reduced relative heart mass. It is probable that this results in a mismatch between cardiac output and the needs of the rapidly growing muscle masses. Romvari et al. [13] reported that the male BUT Big 6 turkeys are disadvantaged in term of the ratio of cardiac stroke volume to body mass. Thereby unfavorable husbandry conditions may lead to cardiovascular disease, because the functional capacity of the heart of heavy turkeys to undertake mild exercise is limited [24]. Linked in with this is a probable less than optimal nutritional and gaseous environment of the animal’s viscus. These factors either together or separately can result in birds being less able to cope with stressors within their living environment.

Because the meat-type and wild-type birds in the present study were housed under identical conditions, the reported morphometric differences are probably due to genetic selection.

However, it has also be taken into account that husbandry conditions may influence the growth of the turkey heart. In order to find the optimal husbandry environments for each turkey line comparative experiments with control groups under different husbandry conditions should be considered.
